# [^18^F]F-Poly(ADP-Ribose) Polymerase Inhibitor Radiotracers for Imaging PARP Expression and Their Potential Clinical Applications in Oncology

**DOI:** 10.3390/jcm13123426

**Published:** 2024-06-11

**Authors:** Honest Ndlovu, Ismaheel O. Lawal, Sipho Mdanda, Mankgopo M. Kgatle, Kgomotso M. G. Mokoala, Akram Al-Ibraheem, Mike M. Sathekge

**Affiliations:** 1 Nuclear Medicine Research Infrastructure (NuMeRI), Steve Biko Academic Hospital, Pretoria 0001, South Africa; ndlovuhonest@gmail.com (H.N.); sipho.mdanda@sanumeri.co.za (S.M.); kgatle.mankgopo@gmail.com (M.M.K.); kgomotso.mokoala@up.ac.za (K.M.G.M.); 2 Department of Nuclear Medicine, University of Pretoria & Steve Biko Academic Hospital, Private Bag X169, Pretoria 0001, South Africa; ismaheel.opeyemi.lawal@emory.edu; 3 Department of Radiology and Imaging Sciences, Emory University, Atlanta, GA 30322, USA; 4 Department of Nuclear Medicine, King Hussein Cancer Center (KHCC), Al-Jubeiha P.O. Box 1269, Amman 11941, Jordan; akramalibrahim@gmail.com

**Keywords:** [^18^F]F PARP inhibitor radiotracers, poly(ADP-ribose) polymerase, homologous recombination, breast cancer-associated genes (*BRCA1/2*), synthetic lethality, poly(ADP-ribose) polymerase inhibitor, PARP inhibitor therapy resistance mechanisms, triple-negative breast cancer, treatment selection, targeted therapy

## Abstract

Including poly(ADP-ribose) polymerase (PARP) inhibitors in managing patients with inoperable tumors has significantly improved outcomes. The PARP inhibitors hamper single-strand deoxyribonucleic acid (DNA) repair by trapping poly(ADP-ribose)polymerase (PARP) at sites of DNA damage, forming a non-functional “PARP enzyme–inhibitor complex” leading to cell cytotoxicity. The effect is more pronounced in the presence of PARP upregulation and homologous recombination (HR) deficiencies such as *breast cancer-associated gene* (*BRCA1/2*). Hence, identifying HR-deficiencies by genomic analysis—for instance, *BRCA1/2* used in triple-negative breast cancer—should be a part of the selection process for PARP inhibitor therapy. Published data suggest *BRCA1/2* germline mutations do not consistently predict favorable responses to PARP inhibitors, suggesting that other factors beyond tumor mutation status may be at play. A variety of factors, including tumor heterogeneity in PARP expression and intrinsic and/or acquired resistance to PARP inhibitors, may be contributing factors. This justifies the use of an additional tool for appropriate patient selection, which is noninvasive, and capable of assessing whole-body in vivo PARP expression and evaluating PARP inhibitor pharmacokinetics as complementary to the currently available *BRCA1/2* analysis. In this review, we discuss [^18^F]Fluorine PARP inhibitor radiotracers and their potential in the imaging of PARP expression and PARP inhibitor pharmacokinetics. To provide context we also briefly discuss possible causes of PARP inhibitor resistance or ineffectiveness. The discussion focuses on TNBC, which is a tumor type where PARP inhibitors are used as part of the standard-of-care treatment strategy.

## 1. Introduction

Non-surgical methods such as chemotherapy and radiotherapy are typically employed in the management of inoperable tumors. These non-surgical methods cause single- and double-strand DNA breaks, either directly or indirectly, which result in tumor cytotoxicity [1]. As part of the DNA damage response (DDR), the cell responds by recruitment of an assortment of proteins and enzymes, amongst other role players, to facilitate the restoration of DNA and cellular integrity [2]. There are various methods for repairing single- or double-stranded DNA damage. Homologous recombination repair (HRR) is one of the mechanisms effecting the repair of double-strand DNA damage, whereas upregulated poly(ADP-ribose) polymerase (PARP) enzymes are key for single-strand DNA damage repair [3,4]. The isoenzyme PARP1 is responsible for >99% of single-strand DNA repair. It is activated during genotoxic stress mainly by single- and/or double-strand DNA breaks [5]. PARP enzymes participate in the DDR by catalyzing the conversion of nicotinamide adenine dinucleotide (NAD+) to polymers of poly(ADP-ribose) (PAR). This “PARylation” of targeted proteins by PARP enzymes culminates in the recruitment of various DNA repair enzymes within nanoseconds of the DNA damage, restoring DNA integrity [6]. Unlike in normal cells, where restoration of DNA integrity is desirable, DNA damage repair equates to treatment resistance/failure in the setting of cancer [7]. This explains the rationale behind the use of PARP inhibitors to “stop” single-strand DNA damage repair mediated by the PARP enzyme complex, as mentioned above. The effectiveness of PARP inhibitors has been linked to the presence of PARP overexpression and homologous recombination repair (HRR) deficiencies [8]. It therefore follows that the presence of both HRR deficiencies and inhibition of PARP enzymes results in the failure of both single-strand and double-strand DNA damage. This phenomenon is referred to as “synthetic lethality” and is commonly seen in triple-negative breast cancer (TNBC) [8,9,10]. Some of these PARP inhibitors in routine clinical use include, Olaparib, Rucaparib, and Talazoparib [11,12,13].

**Figure 1:**
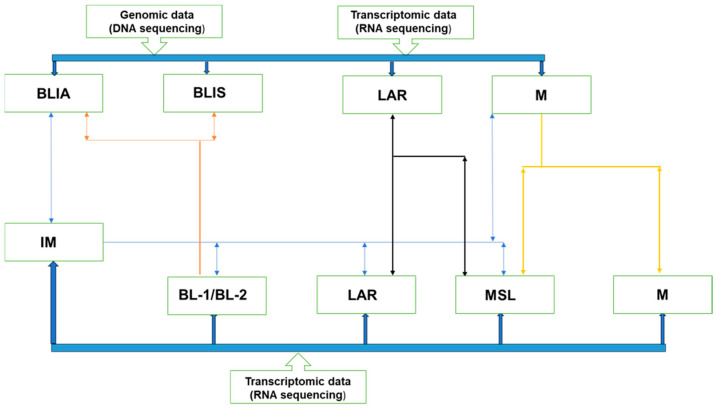
Subtyping of TNBC based on genomic and transcriptomic analysis: Various groups have aimed to classify TNBC using two main systems—genomic and transcriptomic analysis—in isolation or combined. Transcriptomic-only sub-classification divides TNBC into six subtypes, which include IM, BL-1, BL-2, LAR, MSL, and M; while genomic and transcriptomic analysis combined sub-classifies TNBC into four subtypes. These subtypes are BLIA, BLIS, LAR, and M. The systems demonstrate an overlap, which clearly demonstrates that TNBC is a heterogeneous entity.

In TNBC, *BRCA1/2* germline mutation determines PARP inhibitor eligibility. This can be ascertained using BRACAnalysis^®^ or myChoice^®^ (Myriad Genetics, Salt Lake City, UT, USA). These are slowly transitioning from clinical trials to clinical practice [14]. However, the response varies among patients preselected based on *BRCA1/2* germline status; data indicate that between 30% and 70% of these patients do not exhibit favorable responses [15,16]. This implies that there are possibly other factors in addition to homologous recombination defects and *BRCA1/2* mutation status that predict an individual’s response to PARP inhibitors. One possible culprit is tumor heterogeneity. Tumor heterogeneity is the intra-lesional or inter-lesional spatial or temporary variation in phenotypic, somatic, and genomic features [17]. Temporal variations are related to tumor evolution, which may be induced by oncological therapies or environmental factors. These differences in somatic, genetic, and epigenetic changes potentially lead to different responses in different lesions within the same patient [18]. TNBC is a typical example, which is regarded as a homogeneous entity due to the absence of estrogen receptor (ER), progesterone receptor (PR), and human epidermal growth factor receptor-2 (HER2) expression [19]. It is generally associated with *breast cancer-associated gene 1/2* (*BRCA1/2*) germline mutations and PARP upregulation, making PARP inhibitors a feasible treatment option [20,21]. Despite common misconceptions, TNBC is quite diverse, and can be further subdivided according to transcriptomic (RNA) and genomic (DNA) factors. Crucially, these subtypes exhibit shared transcriptome and genetic characteristics that impact TNBC behavior, as outlined below (Figure 1) [22,23,24,25].

Transcriptomic-only sub-classification divides TNBC into six subtypes, which are immunomodulatory (IM), basal-like-1 (BL-1), basal-like 2 (BL-2), luminal androgen receptor (LAR), mesenchymal-like (MSL), and mesenchymal (M). Genomic and transcriptomic analyses combined sub-classifies TNBC into four subtypes, which are basal-like immune-activated (BSLIA), basal-like immune-suppressed (BSLIS), luminal androgen receptor (LAR), and mesenchymal (M). These subtypes demonstrate an overlap, emphasizing the heterogeneous nature of TNBC [26,27,28]. Amongst these subtypes, basal-like constitutes the majority of these TNBC subtypes and is associated with enhanced cell cycles and DNA repair mechanisms, particularly homologous recombination deficiencies and germline/somatic *BRCA1/2* mutations [29]. Other subtypes have upregulation of other pathways. For example, luminal androgen receptor subtype demonstrates upregulation of androgen receptor, ER receptor signaling, and ErB4 signaling despite ER negativity on immunohistochemistry [29]. This implies that the basal-like subtype is most likely to succumb to PARP inhibitor therapy whilst the luminal androgen subtype may succumb to ER-, androgen-, and P13k-targeted therapies [29]. See Table 1 below for an elaborate description of the molecular features, important markers, and feasible therapeutic options for different TNBCs.

Equally important are the intrinsic/primary or acquired/secondary mechanisms of PARP inhibitor therapy [30]. These include restoration of homologous recombination repair and drug efflux. Restoration of homologous recombination deficiency occurs, for example, when a secondary mutation in the affected gene restores the reading frame for the gene [30,31,32]. In tumors overexpressing *Abcb1a* and *Abcb1b* genes, which encode efflux membrane transporter P-glycoprotein (P-gp), the residence time of PARP inhibitors is limited to induce the desired therapeutic effect [5,33,34]. Other mechanisms that have been implicated in PARP inhibitor therapy resistance include epigenetic modification, restoration of ADP-ribosylation (PARylation), and restoration of replication fork protection [31,34].

This supports the idea that understanding PARP overexpression and the pharmacokinetics of PARP inhibitors is crucial when choosing patients to receive PARP inhibitor therapy. Responses observed in patients lacking germline-like mutations in *BRCA1/2* provide additional evidence that PARP upregulation and PARP inhibitor pharmacokinetics are important variables in predicting response to PARP inhibitors. The addition of Olaparib to Bevazucimab in ovarian cancer patients also conferred a significant progression-free survival benefit in patients without homologous repair deficit, according to a comprehensive study conducted by Ray-Coquard et al. [35]. They clarify that the existence of other BRCA-like germline mutations, or “BRCAness”, may be the cause of this [35]. One could argue that since immunohistochemistry shows PARP expression directly, it should also be employed. However, it overlooks tumor heterogeneity and PARPi pharmacokinetics and has a number of confounding variables, such as sampling bias, single lesion, and single time point analysis [36].

The identified limitations in the currently used techniques for assessing PARP upregulation suggest that novel tools are needed for this indication, which may also be useful for studying PARPi pharmacokinetics to quantify drug delivery to tumor lesions and determine the extent of the problem of tumor heterogeneity. PET imaging is a recommendable tool with the potential to fill this gap as it is a robust, noninvasive, sensitive modality capable of providing real-time information on physiological or pathological processes [37]. The recently developed [^18^F]F-radiolabeled PARP inhibitors are the subject of subsequent sections. Despite the existence of other tracers, the discussion will center on fluorinated radionuclides because of their favorable physicochemical features and availability [38]. We will also briefly discuss the potential role of these tracers in treatment selection and as predictive/prognostic tools with a particular focus on TNBC.

## 2. [^18^F]F-Radiolabeled Molecular Probes for PET Imaging

**Figure 2:**
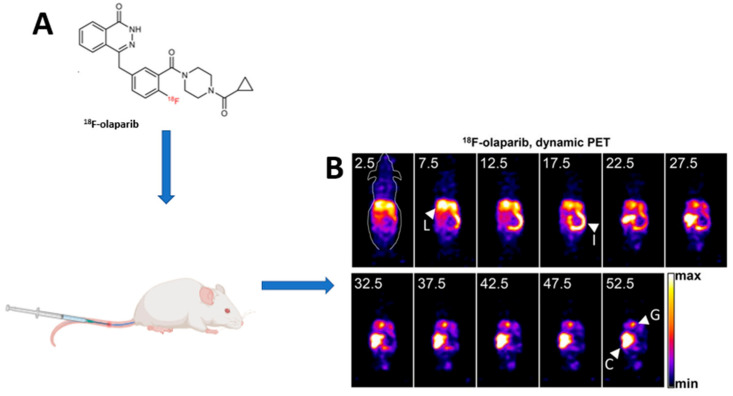
Illustration of PET imaging of PARP expression using [^18^F]F-Olaparib. (**A**) shows the chemical structure of [^18^F]F-Olaparib and (**B**) shows the physiological uptake of [^18^F]F-Olaparib in the liver and gut, whereas uptake seen in C and G is in keeping with tumoral uptake of [^18^F]F-Olaparib consistent with PARP expression.

There is an independent correlation between higher PARP expression and worse outcomes in breast cancer [39]. This implies that knowledge of the in vivo PARP expression/upregulation may be crucial for patient management, especially with PARP inhibitors. Since the PARP1 enzyme is primarily responsible for the majority of the PARP enzymatic activity, efforts to identify in vivo PARP expression have primarily focused on this enzyme [5]. Among these efforts, PET/CT imaging has shown promise as an adjunctive measure in oncology, working in tandem with other traditional diagnostic techniques. An example is the current use of [^18^F]FES PET/CT in breast cancer for estrogen receptor imaging when other diagnostic tests are equivocal amongst other indications [40]. In the case of the in vivo assessment of PARP upregulation, molecular imaging makes use of radiopharmaceuticals which are based on the scaffolding of the FDA-approved PARP inhibitors Olaparib, Rucaparib, and Talazoparib [10,41,42]. Consequently, these characteristics enable an indirect evaluation of the potential biodistribution and pharmacokinetics of PARP inhibitors before they are administered for therapeutic purposes, as illustrated in Figure 2. The [^18^F]F-labeled PET tracers and their associated potential clinical utilities are discussed in subsequent sections below.

### 2.1. Olaparib-Based [^18^F]F-Radiolabeled Molecular Probes

Olaparib, administered orally, is a competitive PARP inhibitor. Its primary site of action is the catalytic site of PARP1 and PARP2 isoenzymes, rendering them non-functional [43]. This results in stalled DNA damage repair and trapping of potentially cytotoxic PARP-DNA complexes [44]. This action of Olaparib synergizes with the DNA-damaging abilities of other antineoplastic regimens. Olaparib is therefore approved as a monotherapy or as part of combinational therapy in various malignancies including TNBC [11]. Therefore, to enhance response to therapy with Olaparib, a radiopharmaceutical should be designed based on its scaffolding. This is also true for other PARP inhibitors. In this section, we discuss the various Olaparib-based radiotracers that have undergone in vitro, preclinical, or clinical investigations.

#### 2.1.1. [^18^F]F-BO

Keliher et al. were amongst the first to develop [^18^F]F-BO (also called [^18^F]F-AZD2281), which is based on the Olaparib scaffolding (AZD2281), using biorthogonal conjugation chemistries [45]. In their preclinical studies, Keliher et al. demonstrated that [^18^F]F-BO accumulation was both sensitive and specific to the PARP1-expressing cells. They noted that the administration of cold/non-radioactive ADZ2281 reduced the [^18^F]F-BO signal/uptake [46]. The findings provided evidence that [^18^F]F-BO is specific to PARP1. Therefore, this implies that when imaged with PET, [^18^F]F-BO could be a useful tool for in vivo assessment of PARP1 upregulation. Another possible application is the quantification of the degree of PARP inhibition during olaparib treatment. In a subsequent study, the same group compared [^18^F]F-BO to the standard of care [^18^F]FDG after PARP inhibitor therapy [46]. They noted little or no change in [^18^F]FDG uptake, whereas the uptake of [^18^F]F-BO decreased. This emphasizes that [^18^F]F-BO outperformed [^18^F]FDG as an imaging biomarker for in vivo PARP upregulation [46,47]. This evidence is promising to support the clinical translation of [^18^F]F-BO PET/CT as a tool for imaging PARP upregulation and therapeutic PARP inhibition. However, this has not been clinically explored to date.

#### 2.1.2. [^18^F]F-PARPi-FL

This is a dual-modality imaging tool developed from an Olaparib scaffolding with [^19^F]F/[^18^F]F isotopic exchange, which makes it capable of PET and optical imaging [46,48]. This tracer has mainly been explored in glioblastomas [49]. Like [^18^F]F-BO, [^18^F]F-PARPi-FL was shown to accumulate selectively in glioblastomas due to high PARP1 expression [50]. In addition, severely reduced uptake of [^18^F]-PARPi-FL was shown in Olaparib-blocked tumors, emphasizing its high specificity [50]. Taking this into consideration, [^18^F]F-PARPi-FL has the potential to assess PARP1 expression for treatment planning and assessing PARP1 blockade in patients on PARP inhibitor therapy in a similar fashion as [^18^F]F-BO [49,50].

#### 2.1.3. [^18^F]F-PARPi

[^18^F]F-PARPi uses the scaffolding of Olaparib by conjugating a 2H-phthalazin-1-one scaffold to 4-[^18^F]fluorobenzoic acid [51]. A notable application of [^18^F]F-PARPi was in brain tumor cell lines and models. Carney and colleagues showed that the in vitro binding capacity of [^18^F]F-PARPi to PARP1 was on a par with that of Olaparib and with good in vivo stability [51]. In their glioblastoma mouse models, [^18^F]PARPi had good pharmacokinetic properties with remarkable in vitro and in vivo stability, as evidenced by minimal defluorination [51]. In addition, the signal-to-noise ratio was favorable, with early washout of tracer from normal tissues. Comparing Olaparib- and non-Olaparib-treated mice, the signal-to-noise ratio was lower in the former, indicating the specificity of [^18^F]F-PARPi for PARP1 [51]. In a patient with glioblastoma treated with laser interstitial thermal therapy, Young et al demonstrated variable [^18^F]F-PARPi uptake within the same tumor [52]. On immunohistochemistry, areas with intense uptake were confirmed to be associated with the tumor while those without, or with low-grade uptake, were associated with treatment-related changes [52]. This shows [^18^F]F-PARPi imaging’s potential to differentiate between viable tumors and treatment-related changes, especially in brain cancer or metastases where standard imaging techniques such as [^18^F]FDG PET/CT have sub-optimal performance [53]. The sub-optimal performance of [^18^F]FDG PET/CT in distinguishing residual tumors from changes induced by treatment is worse with immunotherapy due to the phenomenon of “pseudoprogression”, a phenomenon that also compromises the performance of morphologic imaging techniques for response assessment in this setting [54].

Although other tracers have been used to distinguish viable tumors from treatment-induced changes, [^18^F]PARPi-PET performed better than [^18^F]FET-PET, indicating that [^18^F]F-PARPi may be complementary to amino acid imaging [55]. The same concept has been evaluated with reactive lymph nodes, which may pose a challenge in patients imaged with [^18^F]FDG PET/CT. In their study, Tang et al. demonstrated greater sensitivity and specificity of the [^18^F]F-PARPi in distinguishing inflammatory lymph nodes from active malignancy [56]. The ability of [^18^F]F-PARPi to delineate tumors, especially in areas of high physiological metabolic activity compared to [^18^F]FDG, makes it a potentially valuable tool for radiotherapy planning. This may be helpful in reducing dose to noninvolved tissues contiguous to the tumor during radiotherapy [57,58].

#### 2.1.4. [^18^F]F-Olaparib

Wilson et al. and Bowden et al. developed and refined the semi-automated and automated synthesis of [^18^F]F-Olaparib synthesis with high radiochemical purity [59,60,61]. The same group exposed pancreatic ductal adenocarcinoma models with varying levels of PARP1 expression to [^18^F]F-Olaparib. They showed that [^18^F]F-Olaparib uptake was directly proportional to the level of PARP1 expression [59]. The same group observed reductions in [^18^F]F-Olaparib uptake with a PARP inhibitor blockade in a similar fashion to what was noted with [^18^F]F-BO. They also noted an increase in [^18^F]F-Olaparib uptake with notable increases in PARP1 expression post-radiation in mice tumor models [59]. Chan et al. had similar findings that showed [^18^F]F-Olaparib is mainly governed by PARP expression [62]. These findings warrant validation to determine the optimal level of uptake associated with favorable outcomes on PARP inhibitor therapy. Another important factor was the direct correlation between [^18^F]F-Olaparib and the hypoxia marker EF5, which suggests that hypoxic tissue may show favorable response to PARP inhibitor therapy. However, this correlation remains to be explored in humans [59].

#### 2.1.5. Other Olaparib-Based [^18^F]F PET Radiotracers ([^18^F]FPyPARP; [^18^F]F-20; [^18^F]F-9e; and [^18^F]F-AZD2461)

[^18^F]F-20 is one of the tracers that has been effectively synthesized with good radiochemical yield [63]. Although it has shown some specific PARP binding to glioblastoma and good tumor retention properties, which allowed for preclinical PET visualization of PARP overexpressing tumors, it has associated drawbacks. These include rapid hepatobiliary clearance and in vivo defluorination followed by non-specific [^18^F]F bone tissue uptake in mice [63].

[^18^F]F-9e and [^18^F]F-AZD2461 were developed from AZD2461, which has a similar structure and anticancer activity to Olaparib but is a poor substrate for drug transporters such as P-glycoprotein [64]. This provides an opportunity for assessing in vivo resistance mechanisms in patients unresponsive to PARP inhibitor therapy. This is especially the case in P-glycoprotein-related resistance. The downfall of these tracers was their inability to penetrate the blood–brain barrier. However, this is subject to scrutiny, since in primary or metastatic brain tumors the integrity of the blood–brain barrier is distorted [65]. On another note, [^18^F]F-AZD2461 uptake demonstrated blockade with concurrent AZD2461 administration. No blockade was seen with the co-administration of Olaparib, which makes [^18^F]F-AZD2461 not an ideal tracer if Olaparib is the desired PARP inhibitor for therapy [65].

Stotz et al. developed a less lipophilic variant of [^18^F]PARPi called [^18^F]FPyPARP by exchanging the fluorobenzoyl residue with a fluoronicotinoyl group [42]. This was designed on the basis that [^18^F]F-PARPi and [^18^F]FTT (rucaparib-derived radiotracer discussed later) suffer from hepatobiliary clearance, thereby hampering their use for the detection of abdominal lesions. The authors compared [^18^F]F-PARPi, [^18^F]FPyPARP, and [^18^F]FTT [42]. A partial shift from hepatobiliary to renal clearance of [^18^F]FPyPARP was observed with a decrease in the liver-to-kidney ratio with time. This may facilitate the accurate detection of abdominal lesions [42].

### 2.2. Rucaparib-Based [^18^F]F-Radiolabeled Molecular Probes

Rucaparib (Rubraca™) is an oral, small molecule, and a poly(ADP-ribose) polymerase inhibitor. Its mechanism of action is like that of Olaparib. However, in vitro data suggests that rucaparib is more cytotoxic than Olaparib. This has partly been correlated to the fact that it is a substrate and has the capability of inhibiting P-glycoprotein (P-gp). P-gp is an ATP-binding cassette transporter that extrudes toxins and xenobiotics, which results in reduced efficacy of drugs [13]. However, no clinical evidence exists to suggest differing efficacies to Olaparib, which may be related to poor patient selection [66]. In this section, we delve into the Rucaparib-based [^18^F]F radiotracers that have undergone either in vitro, preclinical, or clinical investigation.

#### 2.2.1. [^18^F]F-FluorThanatrace ([^18^F]FTT)

Zhou et al. showed that among the Rucaparib-based tracers that they had developed and investigated, [^18^F]FTT outperformed others and showed the greatest potential for clinical translation [67]. In both in vitro and preclinical xenograft breast and ovarian cancer mouse models, this and other groups showed a direct correlation between [^18^F]FTT uptake, PARP1 expression, and competitive inhibition with Olaparib co-administration [67,68,69]. Effron et al. also evaluated PARP expression using [^18^F]FTT post-adjuvant PARP inhibitor therapy [70]. Their study provided evidence supporting the relationship between response to PARP inhibitor treatment and the homologous recombination repair deficiency. They observed that tumor cells with homologous recombination repair (HRR) failure and high PARP expression on imaging with [^18^F]FTT post-radiotherapy responded favorably to PARP inhibitor therapy [70]. This is important since it provided the initial evidence that imaging with [^18^F]FTT could predict the response to PARP inhibitor therapy [70]. [^18^F]FTT has been translated clinically in tumors associated with homologous recombination DNA repair deficiencies [71]. Despite an overlap in prostate cancer patients with and without HRR genomic aberrations, median SUVmax was much higher in the former. Importantly, this corroborated with immunohistochemical findings of PARP upregulation, in which patients with high SUVmax values had higher PARP expression on immunohistochemical analysis [72]. This potentially implies that this group may benefit from PARP inhibitor therapy. Additionally, it implies that [^18^F]FTT might be employed as a potential surrogate biomarker for PARP1 expression and as a tool for selecting patients for PARP inhibitor therapy. Its performance compared to screening for *BRCA1/2* mutations in predicting and selecting patients for PARP inhibitor therapy warrants further evaluation [63]. Young et al. studied the pharmacokinetics of [^18^F]FTT and concluded that imaging at 60 min post-injection is optimal, and whole-body SUV is a robust metric for noninvasively quantifying PARP1 expression in vivo [73]. McDonald et al. conducted a two-single-arm prospective non-randomized clinical trial evaluating PARP expression in breast cancer [74]. In their study, PARP expression was assessed in vivo via [^18^F]FTT PET before and after initiation of PARPi treatment. Amongst the four patients included in the preliminary report, the three that had [^18^F]FTT uptake on their pretherapy [^18^F]FTT PET imaging demonstrated resolution of the PET signal on the post-PARP inhibitor therapy PET imaging. Their results indicate that [^18^F]FTT is a potential in vivo tool for determining PARP expression and predicting therapeutic efficacy [74]. The *BRCA1/2* pathogenic variant and non-carriers demonstrated [^18^F]FTT uptake, proving that PARP expression is vital in predicting response to PARP inhibitor therapy. It also demonstrates that [^18^F]FTT PET/CT imaging is a potential surrogate biomarker for PARP expression [75]. There are still several studies investigating the possible use of [^18^F]FTT in ovarian, fallopian tube, and peritoneal breast cancer, and non-ovarian or breast-related solid tumors (NCT03083288, NCT03846167, NCT05226663, and NCT03604315). The objectives include evaluation of changes in [^18^F]FTT uptake measures pre- and post-therapy, and their correlation with PARP1 pathology assays of activity in tissue and hormone receptor status.

#### 2.2.2. [^18^F]F-WC-DZ-F and [^18^F]F-Rucaparib

[^18^F]F-WC-DZ-F and [^18^F]F-rucaparib, which are also based on the rucaparib scaffolding, as analogs of [^18^F]FTT, have been developed and evaluated preclinically [76]. [^18^F]F-rucaparib uptake has shown specificity to PARP expression, evidenced by an increase in uptake correlated with PARP expression in DNA damage in pancreatic cancer models. The tumor kinetics were also favorable, with prompt blood pool clearance [77]. In tumor models treated with [^225^Ac]Ac-PSMA-617, increased uptake of [^18^F]F-WC-DZ-F was noted, in keeping with PARP upregulation as part of the DNA damage response. This demonstrates its potential as a surrogate biomarker for patient selection and predictor of response in patients with or without PARP inhibitor therapy as an adjuvant to peptide radioligand therapy, which remains to be explored clinically [78].

### 2.3. Talazoparib-Based and Other [^18^F]F-PARP Inhibitor Molecular Probes

Talazoparib was first approved in combination with enzalutamide for homologous recombination repair (HRR) gene-mutated metastatic castration-resistant prostate cancer (mCRPC) [79]. It is a potent PARP (including PARP1 and -2) inhibitor. Selective anti-tumor activity was seen in vitro in *BRCA1*-, *BRCA2*-, and *PTEN*-deficient breast cancer models [80]. Preclinical and clinical studies have demonstrated its potential for use as a mono- or combinational therapy in various tumors [79].

#### [^18^F]F-Talazoparib

[^18^F]F-talazoparib has been developed on a scaffolding of Talazoparib, and it has demonstrated good tumor uptake with similar results to other PARP inhibitor radiolabeled tracers [81]. Shuhendler et al. explored [^18^F]F-SuPAR, which is a radioactive fluoronicotinamide adenine dinucleotide (NAD) analogue recognized by PARP1/2 and incorporated into the long-branched polymers of poly(ADP-ribose) (PAR) [82]. They were able to map the dose- and time-dependent activation of PARP1/2 following radiation therapy in breast and cervical cancer xenograft mouse models with [^18^F]F-SuPAR. They also noted that tumor PARP1/2 response to therapy was determined by [^18^F]F-SuPAR PET within 8 h of administration of a single dose of radiation, equivalent to one round of stereotactic ablative radiotherapy [82].

## 3. Discussion

Selection for PARP inhibitor is usually based on *BRCA1/2* germline mutation status. However, the variable responses to PARP inhibitors which have been attributed to tumor heterogeneity, and intrinsic and acquired resistance suggest other factors that need to be considered. These factors include knowledge of PARP expression and tumor handling/pharmacokinetics of PARP inhibitors. This obviates the need for adjunct in vivo assessment tools capable of assessing whole-body PARP expression and predicting PARP inhibitor pharmacokinetics, such as PET/CT imaging. In this article, we reviewed [^18^F]F-radiolabeled PARP inhibitor radiotracers for imaging PARP1/2 overexpression in various cancers. Both preclinical and clinical studies have demonstrated the sensitivity and specificity of these radiotracers to PARP expression. The fact that the structure of these radiotracers is based on the scaffolding of the approved PARP inhibitors also provides a unique opportunity for predicting the pharmacokinetics of PARP inhibitors prior to their administration, reducing futile administration of these expensive PARP inhibitors. It so follows that the choice of the radiotracers should have a similar structure to the intended PARP inhibitor regimen. This is especially important when there is a suspicion of expression of P-glycoprotein by the tumor in which case Rucaparib, a PARP inhibitor which inhibits P-glycoprotein, may be the favorable option. Clinical studies performed to date have shown the potential of these radiotracers to assess early response to PARP inhibitor therapies, and distinguish between post-treatment/reactive lymph nodes and active tumors due to their predilection for active tumors. This review focused on [^18^F]F-radiolabeled PARPi (see Table 2) due to their robust synthesis, their inherent physical properties, and the wide availability of [^18^F]F making them the most feasible choice for the clinic.

Other PET imaging agents labeled with [^11^C]C, [^64^Cu]Cu, and [^68^Ga]Ga have been used in animal models [83,84,85]. Although [^68^Ga]Ga-labeled radiotracers may be advantageous due to the in-house production of [^68^Ga]Ga from [^68^Ge]Ge/[^68^Ga]Ga generators compared to their [^18^F]F-labeled counterparts, [^68^Ga]Ga has a relatively short retention time and low uptake levels, the latter of which may be attributed to their less lipophilic nature, reducing their ability to cross lipophilic cell membranes and bind to PARP [83,86]. On the other hand, with [^11^C]C its short half-life and production from a cyclotron may affect distribution compared to [^18^F]F [87]. [^64^Cu]Cu-radiolabeled tracers may offer an added advantage due to the longer half-life of [^64^Cu]Cu allowing for delayed imaging and superior target-to-background ratios [88]. However, these have not yet been explored clinically or compared to [^18^F]F-labeled PARP inhibitor radiotracers.

## 4. Conclusions

Heterogeneous responses to PARP inhibitors attributed to tumor heterogeneity and the resistance mechanisms to PARPi justify the need for imaging PARP expression and PARP inhibitor pharmacokinetics for patient selection to complement *BRCA1/2* analysis. The [^18^F]F-radiolabeled PARP inhibitors, which have undergone substantial preliminary clinical research over the past decade, have found a good place in this regard due to their robust synthesis, their inherent physical properties, and the wide availability of [^18^F]F. They have shown promise in patient selection, predicting and monitoring response to PARP inhibitor therapy, and distinguishing tumors from reactive lymph nodes/post-treatment changes.

## 5. Future Perspectives

Tumor heterogeneity, which entails inter and/or intra-lesional variability in genomic and phenotypic features within a lesion and its metastases, has been implicated as a probable cause of variable response to PARPi. Other possible resistance mechanisms, with an emphasis on TNBC, have been discussed in this review. As discussed in this review, [^18^F]F radiotracers have shown promise in addressing this gap, as discussed in this review, have shown promise in addressing this gap. This is because of their correlation to PARP expression and the ability to image in vivo PARP inhibitor pharmacokinetics. Potential scenarios for PARP imaging or aspects that may be potentially useful or need to be explored include:

- Assessing PARP expression status in lieu of biopsy in lesions. This could be in lesions that are inaccessible to biopsy or in suspected tumor heterogeneity, especially in patients with metastatic disease.
- At the time of initial diagnosis of primary or metastatic disease when considering PARP therapy.
- Detecting PARP expression when other imaging tests are equivocal or suspicious. This may also include patients with other immunohistochemical subtypes that may have *BRCA*-like mutations. *BRCA1/2* mutations, PARP expression, and uptake of [^18^F]FTT have been seen even in patients with hormonal receptor expression. Should conventional therapies fail, the addition of PARP inhibitor therapy may be considered.
- PARP inhibitor therapeutic monitoring. This includes early assessment of adequate blockade of PARP.
- Differentiating therapy or inflammation-related findings from malignancy in which [^18^F]FDG may not be able to differentiate. This also includes better delineation of tumor from adjacent inflammation or areas with intense metabolic activity, in which [^18^F]FDG performs dismally.
- Evaluating patients for eligibility with PARP-targeted radioligand therapies [41,89,90,91].
- Assessing in vivo resistance mechanisms in patients unresponsive to PARP inhibitor therapy, especially P-glycoprotein-related resistance, despite confirmed PARP upregulation on immunohistochemistry.
- Establishing the optimal scan modality (static vs. dynamic), timing, and other technical aspects to better address the value of PARP inhibitor imaging in various clinical scenarios.
- Evaluation of the added value of PARP inhibitor PET/MRI.
- Development of less lipophilic PARP inhibitor tracers to facilitate the detection of PARP upregulation in hepatic and abdominal lesions. This would require the identification of a specific carrier which would allow the hydrophilic molecule to cross the cellular membrane since PARP is located within the nucleus.
- Correlation of PARP expression based on validated IHC scoring criteria and the semi-quantitative evaluation of PARP expression on PET-based imaging.
- Determining the prognostic and predictive role of PARP inhibitor PET/CT imaging using semi-quantitative parameters in patients being worked up for PARP inhibitor therapy.

**Table 2:** Summary of [^18^F]F Poly(ADP-ribose) polymerase inhibitor-based radiotracers and their respective potential applications.

Scaffolding	Radiotracer	Preclinical/Clinical (P/C)	Pertinent Conclusion(s)
Olaparib	[^18^F]F-BO	P	Development of [^18^F]F-BO from Olaparib scaffolding with high-yield fluorination technique [45]. Uptake in PARP1-expressing tumor cell lines in in vivo mice models, and inhibition of uptake by cold Olaparib (AZ2D2281). In comparison with [^18^F]FDG, [^18^F]F-BO showed early changes post-PARP inhibition compared to [^18^F]FDG [46,47].
	[^18^F]F-PARPi-FL	P	Development of [^18^F]F-PARPi-FL [48]. Use in glioblastoma tumor models and xenografts. Specificity to PARP1 expression demonstrated after correlation with immunohistochemistry. Possible application in treatment planning and assessment of PARPi blockade [49,50].
	[^18^F]F-PARPi	P/C	Development and improvement of the synthesis of [^18^F]F-PARPi from Olaparib by conjugating a 2H-phthalazin-1-one scaffold to 4-[^18^F]fluorobenzoic acid [51]. [^18^F]F-PARPi images tumor heterogeneity [52]. [^18^F]F-PARPi uptake is specific to tumor uptake [52,55,92]. Target engagement and potential for monitoring PARP1 blockage by PARPi [92]. Discriminating radiation injury from the recurrent tumor with [^18^F]PARPi: Comparison with [^18^F]FET [55]. Differentiating malignant from inflamed lymph nodes [56]. Phase I/2 study in head and neck tumors: Comparison with [^18^F]FDG [57]. Better performance of [^18^F]F-PARPi in detecting primary tongue tumors: Comparison with [^18^F]FDG [58].
	[^18^F]F-Olaparib	P/C	Development and refinement of [^18^F]F-Olaparib production with high radiochemical yield [59,60,61]. [^18^F]F-Olaparib uptake proportional to PARP1 expression. Effective blockade seen post-PARP inhibition; can be used to assess therapeutic efficacy. Increased uptake as a DNA damage response post-irradiation. (In some cases PARPi may be more effective post-irradiation.) [^18^F]F-Olaparib uptake correlated to ER5 hypoxia marker; therefore, this may be effective in hypoxic tissue [59]. Tumor uptake of radiolabeled PARP inhibitors, such as [^18^F]F-Olaparib, is governed by more than PARP expression levels alone [62].
	[^18^F]F-20	P	Synthesis of [^18^F]F-20. Specific uptake in PARP-expressing tissue or cells. Problematic hepatobiliary excretion and in vivo defluorination leads to non-specific [^18^F]F bone uptake [63].
	[^18^F]F-9e and [^18^F]F-AZD2461	P	Synthesis of [^18^F]F-9e and [^18^F]F-AZD2461. Non-blood barrier permeants. [^18^F]F-AZD2461 uptake not affected by Olaparib blockade; therefore, cannot be used in cases of Olarapib blockade [64,65].
	[^18^F]FPyPARP	P	Less lipophilicity with less hepatobiliary excretion. Favorable for theranostic translation [42].
Rucaparib	[^18^F]FTT	P/C	Development of [^18^F]FTT. Increased specificity for PARP1, competitive inhibition with Olaparib for PARP [67]. PARP expression and activity correlates to [^18^F]FTT uptake in vitro and in a xenograft model of breast and ovarian cancer [68,69]. [^18^F]FTT uptake a surrogate predictor of response to PARP inhibitor adjuvant therapy to radiation [70,72]. Higher SUVs in HRR than non-HRR but overlap was noted [72]. SUVs at 60 min a robust metric for noninvasively quantifying PARP1 expression in vivo [73]. [^18^F]FTT is a predictive and pharmacokinetic biomarker which can be used for patient selection for PARPi [74,75]. Evaluating in vivo PARP1 expression with [^18^F]FTT PET/CT in primary or recurrent breast cancer (NCT03083288; NCT03846167; NCT05226663 and NCT03604315). Serial imaging of the novel radiotracer [^18^F]FTT by PET/CT (NCT03604315). Summary of preclinical to clinical progress of [^18^F]FTT [71].
	[^18^F]F-rucaparib	P	Uptake correlated to PARP expression [77].
	[^18^F]F-WC-DZ-F “aka” [^18^F]F-PARPZ	P	Uptake correlated to PARP expression. Increased uptake correlated to PARP expression as a DNA damage response post-[^225^Ac]Ac-PSMA-11 therapy [78].
Talazoparib	[^18^F]F-talazoparib	P	Development and uptake correlating to PARP expression [81].
Other	[^18^F]F-SuPAR	P	Prediction of response. Development and uptake correlating to PARP expression [82].

## Figures and Tables

**Table 1:** Molecular features, important markers, and feasible therapeutic options for different TNBC subtypes.

Subtype		Molecular Features	Important Markers	Possible Therapies
Basal-like (BL)	BL-1	Elevated cell cycle and DDR gene expression.	*ATR/BRCA*, Ki-67	Cisplatin and PARP inhibitors
	BL-2	Enriched in growth factor signaling, metabolic pathways, and myoepithelial signaling.	EGFR, IGF1R, NGF, MET, Wnt/b-catenin, EPHA2, TP63	Growth factor inhibitors
Immune enriched	IM and/or BSLIA	Genes involved in immune and cytokine signaling transduction pathways.	IL-12, IL-7, NFKB, TNF, JAK/STAT	Immune check point inhibitors
Mesenchymal	Mesenchymal	Gene expression for EMT, cell motility, and differentiation.	Wnt, ALK, TGF-β	
	Mesenchymal-like	Increased growth factor signaling compared with (M), low proliferation, enrichment of genes associated with angiogenesis and stem cells, and low claudin expression.	EGFR, PDGF, ERK1/2, TGF-β, Wnt/β-catenin	EGFR, PDGF, ERK1/2, TGF-β inhibitors, growth factor inhibitors, Src inhibitors
Luminal androgen receptor (LAR)	LAR	Increase in hormonally regulated pathways, AR signaling, and high rate of PIK3C-activating mutations.	AR, FOXA1, KRT18, XBP1, and ESR1	AR antagonists, PI3K inhibitors, Hsp inhibitors, ER pathway inhibitors

ALK, anaplastic lymphoma kinase; ATR, ataxia telangiectasia and Rad3-related protein; EGFR, epidermal growth factor receptor; EMT, epithelial–mesenchymal transition; EPHA2, EPH receptor A2; ERK, mitogen-activated protein kinase; FOXA1, forkhead box A1: Hsp90, heat shock protein 90; IGF1R, insulin-like growth factor 1 receptor; IL, interleukin; JAK, Janus kinase; MET, hepatocyte growth factor receptor; mTOR, mechanistic target of rapamycin; NF-kB, nuclear factor-kappa B; NGF, nerve growth factor; PARP, poly(ADP-ribose) polymerase; PD-1, programmed cell death 1; PDGF, platelet-derived growth factor; PD-L1, programmed cell death 1-ligand 1; PI3K, phosphoinositide 3-kinase; PIK3CA, phosphatidylinositol-4,5-bis-phosphate 3-kinase catalytic subunit alpha; Src, SRC proto-oncogene; STAT, signal transducer and activator of transcription; TGFβ, transforming growth factor beta; TNF, tumor necrosis factor; Wnt, Wnt family member; XBP1, X-box binding protein 1 (adapted from Lehman et al. [27]).

## Data Availability

The articles quoted and referenced are available online as referenced. The images used for the review are available from the corresponding author on request.
